# Gamble While You Gamble: Electronic Games in Ontario Charitable Gaming Centres

**DOI:** 10.1007/s11469-015-9557-y

**Published:** 2015-05-01

**Authors:** Kevin Harrigan, Dan Brown, Vance MacLaren

**Affiliations:** Gambling Research Lab, University of Waterloo, 200 University Ave. West, Waterloo, ON Canada N2L 3G1; School of Computer Science, University of Waterloo, Waterloo, ON Canada; Brandon University, Brandon, MB Canada

**Keywords:** Problem gambling, Electronic gambling machines, Bingo, Charitable gaming

## Abstract

Electronic Bingo games have recently appeared in Ontario Charitable Gaming Centres. Here we summarize the characteristics of this novel form of electronic gambling, and give a detailed characterization of one game. We contend that these games have structural characteristics that make them similar to modern Electronic Gaming Machines (EGMs) that feature multiline slots games. These features include a fast and continuous gaming experience, with player adjustable win size and reinforcement rate, a high frequency of losses disguised as wins, and highly salient near misses. Some of these games also have bonus rounds and provide players with a list of recent wins. We conclude that provincial and state gaming authorities should be aware that the placement of Bingo EGMs in existing Bingo facilities may increase problem gambling among an already well-established community of Bingo enthusiasts.

Charities in Canada often raise funds using Bingo events, raffles and instant win ticket lotteries. Our home province of Ontario has experienced a dramatic drop in the popularity of its charitable gaming sector in recent years. In 2002 there were approximately 230 bingo halls helping to support nearly 6000 charities, but as of 2012 this had dropped to just 61 halls supporting 2800 charities (OLG 2012). There are several likely contributors to this decline, including a smoking ban, a stale product that did not change much between 2002 and 2012, and competition from newer forms of gambling, including Electronic Gaming Machines (EGMs) at casinos and slots-at-racetracks. These bingo halls are owned and operated by private companies, with a portion of profits going to the charitable organizations that volunteer at the halls. The Criminal Code of Canada allows these charitable bingo halls with oversight from provincial regulators, as long as they do not have EGMs. In Ontario, the regulator is the Alcohol and Gaming Commission of Ontario (AGCO). As part of a plan to modernize gambling in Ontario (OLG 2012), the Ontario Lottery and Gaming Corporation (OLG) has spearheaded the introduction of electronic gambling into the bingo halls, and rebranded them as Charitable Gaming Centres. One crucial aspect of this modernization is the absorption of the electronic games at Ontario Charitable Gaming Centres into the operational mandate of OLG as only OLG can conduct and manage EGMs in Ontario.

Previous research has described how this development has allowed paper instant win tickets to now be sold in Charitable Gaming Centres using Video Instant Ticket Vending Machines that have an appearance and style that is essentially identical to multiline slot machines (MacLaren et al. 2015b). In the present paper we describe electronic Play on Demand (POD) Bingo games that have features similar to multiline slots games. We believe that the modernized charitable gaming sector should be carefully monitored as a potential source of increased problem gambling. We also caution other jurisdictions that may be considering introduction of these games to view them as significantly different from the paper-based ancestors of their new electronic counterparts. Indeed, we believe that these Bingo EGM games have features that are more akin to slots EGM games which are well known for their high risk of harm to consumers (Dow Schüll 2012; Dowling et al. 2005).

To explain the basis for these conclusions we will begin by briefly reviewing evidence that some features of modern EGMs may be thought of as the ‘active ingredients’ in these potentially addictive products. Use of that pharmacological term is intended to suggest a demonstrable relationship between the presence of those features and heightened potential for behavioral addiction in all electronic games that have them, including slots, video poker, and now electronic Bingo. We then describe the nature of traditional Bingo games, followed by a detailed description of one example of an electronic version of the game with features that parallel the most problematic aspects of multiline slots games.

## The Active Ingredients in Addictive Slots Games

Gambling researchers have learned a great deal in recent years about the nature of Gambling Disorder (Potenza 2013, 2014), as well as the features of EGM games that increase their potential for revenue generation at the cost of higher risk of problem gambling among some players (Dixon et al. 2015b). Gambling Disorder is unique among the addictive syndromes in the DSM-5 (American Psychiatric Association 2013) in that there is no exogenous pharmacological agent responsible for the atypical brain function and behavior of problem gamblers. Nevertheless, altered anticipatory reward processing has been demonstrated in experiments using behavioral (Dixon et al. 2013), psychophysiological (Dixon et al. 2011), and neuroimaging indicators (Clark 2014) of responses to near-miss outcomes in slots games. Near-miss outcomes during gambling are non-win outcomes that appear to have almost achieved a large prize win. Importantly, near miss effects have been shown both in problem gamblers and in cocaine addicts compared to healthy controls (Worhusky et al. 2014). An even more intriguing finding is that even within a single play session there is a shift in activation away from brain areas underlying consummatory aspects of reward, and toward the areas evoked by reward anticipation, and that this shift is moderated by trait impulsivity (Shao et al. 2013). The shift toward “wanting” at the expense of “liking” is a classic feature of alcohol and opiate addiction (Robinson and Berridge 1993), and a chronic decline in mood may form an ‘allostatic load’ that supports escapist addictive behavior through negative reinforcement (Koob 2013). Thus it is not surprising that the impulsive and negative affective personality traits that are commonly seen in problem gamblers (MacLaren et al. 2011) may increase the likelihood of problem gambling among EGM players by increasing their use of gambling as a way of escaping negative emotional states (MacLaren et al. 2015a). The repetitive and continuous nature of EGMs may be a critical attraction for so-called ‘escape gamblers’.

Cognitive distortions like the gambler’s fallacy and illusion of control are typical of problem gamblers (Goodie and Fortune 2013), and it is possible that the design of multiline slots games may promote these distorted beliefs. Although the long-term proportion of wagers that are returned to players in the form of prizes is always less than 100 % and cannot be altered in any way by players (Harrigan et al. 2011), modern EGMs allow players to control the number of simultaneous wagers they can make on each spin as well as the size of those wagers. These controls effectively allow them to purchase a higher frequency of winning outcomes and nominally larger prizes, despite no change in the hold or payback percentage of the game (Harrigan et al. 2014). The effect of apparent wins increasing in frequency as the number of played paylines increases, is largely due to outcomes where the total amount won is less than the total amount that was bet on all of the simultaneous gambles made on the spin. Such Losses Disguised as Wins (LDW) are cognitively misinterpreted as winning outcomes (Jensen et al. 2013) and evoke psychophysiological arousal similar to legitimate wins (Dixon et al. 2010). Players prefer frequent wins and less volatile play sessions (Templeton et al. 2014), and experienced players know that the ‘lines wagered’ and ‘bet per line’ parameters can be adjusted to increase the frequency and size of wins (MacLaren 2015). Players who feel skilled at playing these games might have the greatest anticipation of reward because of the illusion that they can play optimally (Harrigan et al. 2014). The combination of continuous play, with highly memorable jackpots and bonus rounds presented amongst losses, near misses, LDWs and small wins in a random ratio schedule (Haw 2008) is a recipe for promoting hope of imminent reward over an extended gaming session. All of these features play into the distorted cognitions and motives of problem gamblers.

## The Context of Playing POD Bingo Games

Patrons of Ontario Charitable Gaming Centres can participate in up to five forms of gambling. First, they can play traditional Bingo with numbers randomly drawn and called out while players dab the paper sheets they purchased for the game. If they wish to play more Bingo cards than they can manage manually, they can also use the POD terminals located on their tables to play up to 12 virtual cards. The player can either dab these virtual cards using the touchscreen, or press the “dab” button to have them all dabbed automatically. If the player wins on one of the electronic cards, then the screen flashes the word *Bingo* and the player yells “Bingo!” to receive their prize. After confirmation of the win by a staff member, the winning amount is electronically added to the player’s balance on the terminal. A third way to play Bingo is the POD games described below, which are always available whether or not a live Bingo session is being called. The fourth option is to buy paper instant win lottery tickets, and the fifth is to play electronic versions of the instant tickets on the POD terminals or on Video Instant Ticket Vending Machines that are accompanied by slots-like audiovisual feedback. Some of these types of gambling can be done simultaneously or between live Bingo sessions.

To play regular live Bingo the player typically purchases a package of paper cards for the session. To play on the POD terminal, the player purchases a voucher at the same counter where the paper cards are purchased. The player then takes the voucher with its current balance to any POD terminal and enters the voucher number and a user-chosen password. The terminal maintains the player’s balance and the player can log out at any time and then use that same voucher later at the same terminal or at a different terminal. At the end of play a winner can redeem the voucher at the ticket counter by entering their password. This system is very similar to the “ticket in ticket out” system used on the slots floor of modern casinos.

## A Summary of POD Games

In this section we briefly describe some of the POD games we have observed and played in Ontario Charitable Gaming Centres. There were five POD games in the Charitable Gaming Centres that we visited most frequently, created by eQube Gaming (www.eqube.com). We played these five games for approximately 40 h.

- ***Lucky Clover Bingo***: A bingo game with a traditional 5 × 5-square card with the middle square “free.” Twenty-four numbers are always called. The player wins something if the result on the card matches one or more of 53 winning patterns. The player can play from one to four cards at a time. The game is very fast, as all 24 called numbers are played automatically by the computer on all of the cards in approximately 2 s, after which any payout is added to the player’s balance.
- ***Old Glory Bingo***: A bingo game that has a screen layout that is very similar to Lucky Clover but with a different set of winning patterns, a smaller maximum prize, and it is slower.
- ***The Aztec Game***: A game with a 5 × 5 grid of symbols. The game has 12 lines (5 horizontal, 5 vertical, and 2 diagonal) upon which the player can wager. The player wins if five identical items are lined up on a played line.
- ***Treasure Island***: An electronic version of the paper Bingo-themed scratch ticket games that are sold in Ontario convenience stores and lottery kiosks.
- ***Berri Fruiti***: An electronic version of the paper slots-themed break open tickets that are sold in Ontario convenience stories and lottery kiosks. Break open tickets are also commonly known as “Nevada tickets” or “pull tabs.”

Using information freely distributed in a printed brochure about the POD games that we obtained at a Charitable Gaming Centre, plus some information given in response to our request from OLG, we collated some key parameters of these games and summarized them in Table 1. For example, in the *Aztec Game* the minimum bet is $0.05 on one line and the maximum is $3.00 with $0.25 wagered on each of 12 lines. A player wagering on one line wins something on 8.56 % of the plays, and loses an average of 15.5 % of their wager on each play. The game can be played approximately 15 times per minute so if a person plays the game continuously they will lose an average of $0.12 with the minimum bet, or $6.98 per minute with the maximum bet. ($3.00 × 15.5 % × 15).

**Table 1:** A comparison of POD games

Name	Bet/card		# Cards						Loss per minute	
	Min	Max	Min	Max	Hit%	Hold%	Jackpot	Plays/Min	Min	Max
Lucky clover	$0.25	$1.00	1	4	16.44 %	8.90 %	$10,000	30	$0.67	$10.68
Old glory	$0.25	$1.00	1	4	16.41 %	9.68 %	$6,000	10	$0.24	$3.87
Aztec	$0.05	$0.25	1	12	8.56 %	15.50 %	$250	15	$0.12	$6.98
Treasure Isl.	$0.50	$0.50	1	2	22.35 %	15.20 %	$200	10	$0.76	$1.52
Berri Fruiti	$0.50	$0.50	1	1	19.21 %	25.00 %	$500	10	$1.25	$1.25

In some Charitable Gaming Centres we observed and played other games that were similar to the previous games, and were created by Multimedia Games (www.multimediagames.com). We played these games for approximately 10 h. These games are called *Fill’er Up, High Striker Bingo, Bingo Latte, High Sticks Bingo, Presto Bingo,* and *Tropical Treasures Bingo.* Some notable differences are that some of these games have much higher jackpots, with *Tropical Treasures Bingo* having the highest jackpot at $115,000.00. These POD Bingo games also allow the player to play up to nine Bingo cards at once, as compared to *Lucky Clover* and *Old Glory* which only allow the player to play four cards. Some of these games also have bonus rounds.

## A Detailed Analysis of Lucky Clover

**Fig. 1:**
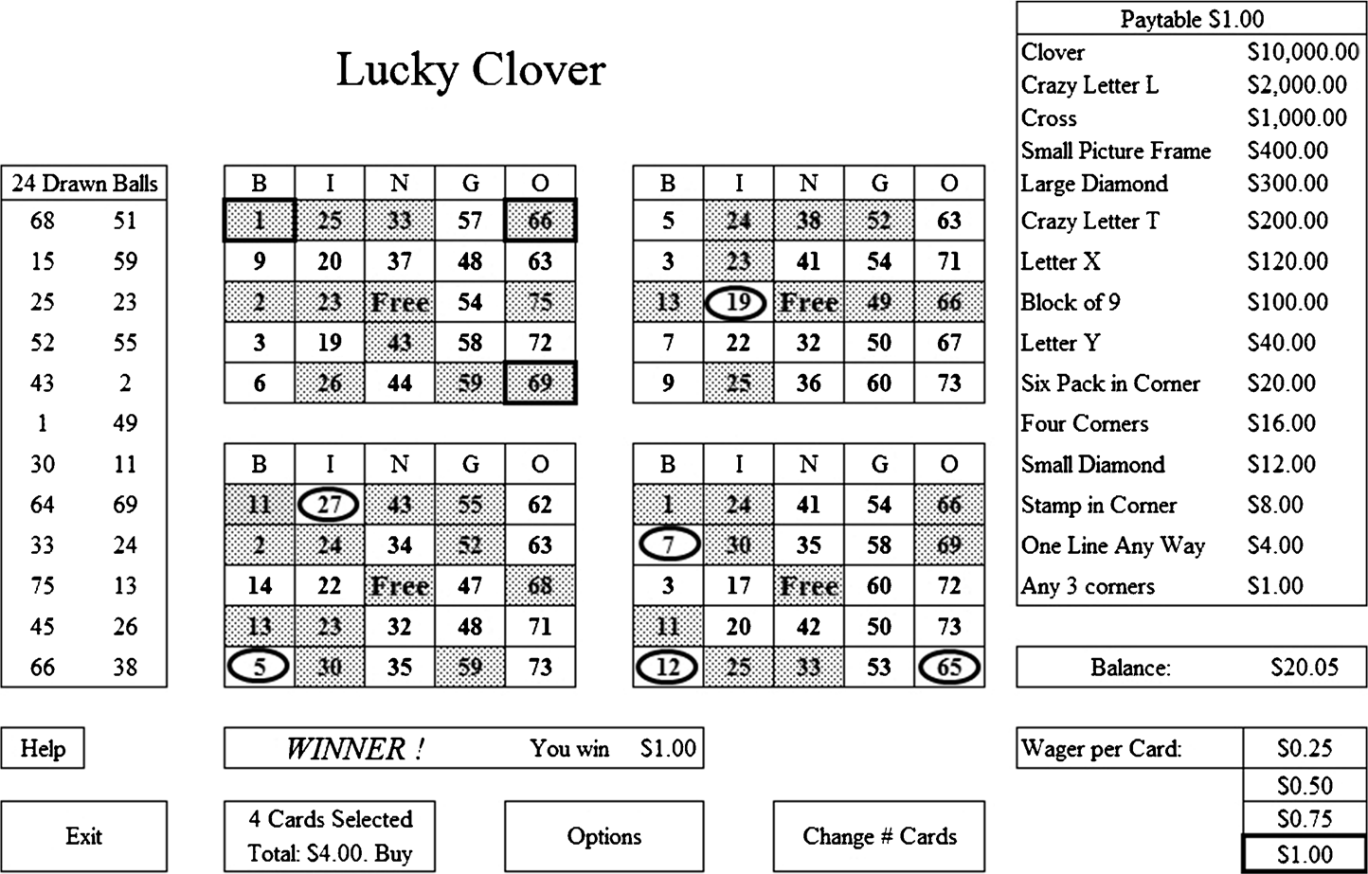
Schematic of the screen for the game *Lucky Clover*

In this section we describe one POD game, Lucky Clover, to give the reader an in-depth look at a POD game. A schematic of the computer touchscreen for *Lucky Clover* is shown in Fig. 1. The game is based on North American Bingo, where each card has 25 squares including the ‘free’ middle square. Under the “B” the numbers are 1–15, under the “I” they are 16–30, under the “N” they are 31–45, under the “G” they are 46–60, and under the “O” they are 61–75. The patron plays from one to four cards at a time, which are shown in the middle of the screen. In Fig. 1 the player is playing four cards and can wager $0.25, $0.50, $0.75, or $1.00 per card. In Fig. 1 the player is wagering $1.00 per card, for a total wager of $4.00.

There are 15 winning patterns, named in the paytable on the right-hand side of the screen in Fig. 1. If one or more of the 15 winning patterns is achieved, the prize corresponding to the highest paying pattern is awarded. Many of the 15 patterns can occur in multiple places on the card. For example, the pattern “One Line Any Way” occurs 12 times (i.e., 5 horizontal lines, 5 vertical lines, and 2 diagonal lines much like the *Aztec Game*). In total there are 53 different ways the 15 patterns can be arranged, and thus there are 53 possible ways of winning. The maximum prize per card is $10,000, which is achieved when the player has wagered $1.00 on a card and has covered all 12 squares of the highest paying “Clover” pattern.

After the player selects the number of cards and wager per card, the player presses the button labelled “4 Cards Selected—Total $4.00—Buy” to initiate the game. There is no button labelled “Play”. As described in the POD brochure, “The cash display on the lower right section of the screen shows your current balance, which increases with winnings and decreases with purchases.”. Using this terminology, expenditures are not referred to as losses but rather as the purchase price of playing, and so any outcome can be presented either as a “non-win” or a “win”.

When play is initiated, 24 random numbers are drawn by the computer and virtually dabbed on all cards in approximately 2 s. The 24 drawn balls are shown on the left-hand side of the screen.

Here we explain the outcome of each of the four cards shown in Fig. 1.

- The top left card has 12 squares dabbed including the numbers 1, 66, and 69, which form an “Any 3 Corners” win that pays $1.00. In *Lucky Clover*, any winning pattern is highlighted in green (these are shown with thick borders in Fig. 1).
- The upper right card has nine numbers dabbed, but none are part of a winning pattern. In *Lucky Clover*, any winning patterns that are missed by one square have that missed square shown in flashing red (these are shown as circles in Fig. 1). For example, in the upper right card, the number 19 is displayed in flashing red to indicate that the horizontal line formed by 13, 19, Free, 49, and 66 would have been a “One Line Any Way” win if the number 19 had been picked.
- The bottom left card has 12 numbers dabbed, but no win. It has two squares in red: the 27 in the top left to indicate that the player just missed the “Stamp in Corner” and the 5 in the bottom left to indicate the “Stamp in Corner” in the bottom left was just missed.
- The card in the bottom right has nine numbers dabbed, and no wins. It has the number 7 in red to indicate there would have been a “Stamp in Corner” win if the number 7 had been called. It has the numbers 12 and 65 in red to indicate that an “Any 3 Corners” win would have occurred if either 12 or 65 had been called.

The result of the play in Fig. 1 is that there are winning sounds, the winning card flashes, the winning pattern flashes, and the screen displays “WINNER! You win $1.00.” The player has wagered $4.00 and “won” $1.00—even though the net loss of that play is actually $3.00. In reality the player has not won a profitable prize; what they got was an LDW and six near misses.

Table 2 provides the paytable for *Lucky Clover,* which was provided to us by OLG. Table 2 shows that the player has a 0.000025 % probability of winning the jackpot of $10,000. In gambling, when the win is equal to the wager it is called a “push.” Table 2 shows that the probability of getting a push when wagering a single card is 7.19 %. We have calculated the column “Plays per Win,” which shows that when playing one card the player wins something, on average, on every 6.08 plays.

**Table 2:** Lucky clover paytable

		Amount wagered and wining prizes				Hit Freq.	Plays/Win
		$0.25	$0.50	$0.75	$1.00		
1	Clover	$2,500.00	$5,000.00	$7,500.00	$10,000.00	0.000025 %	4,000,000.00
2	Crazy letter L	$500.00	$1,000.00	$1,500.00	$2,000.00	0.001307 %	76,511.09
3	Cross	$250.00	$500.00	$750.00	$1,000.00	0.000903 %	110,741.97
4	Small picture Fr.	$100.00	$200.00	$300.00	$400.00	0.002713 %	36,859.57
5	Large diamond	$75.00	$150.00	$225.00	$300.00	0.005752 %	17,385.26
6	Crazy letter T	$50.00	$100.00	$150.00	$200.00	0.004222 %	23,685.46
7	Letter X	$30.00	$60.00	$90.00	$120.00	0.008177 %	12,229.42
8	Block of 9	$25.00	$50.00	$75.00	$100.00	0.021534 %	4,643.82
9	Letter Y	$10.00	$20.00	$30.00	$40.00	0.069884 %	1,430.94
10	6 pack in corner	$5.00	$10.00	$15.00	$20.00	0.373995 %	267.38
11	Four corners	$4.00	$8.00	$12.00	$16.00	0.885920 %	112.88
12	Small diamond	$3.00	$6.00	$9.00	$12.00	0.616968 %	162.08
13	Stamp in corner	$2.00	$4.00	$6.00	$8.00	3.113716 %	32.12
14	1 line any way	$1.00	$2.00	$3.00	$4.00	4.144289 %	24.13
15	Any 3 corners	$0.25	$0.50	$0.75	$1.00	7.193428 %	13.90
				Hit frequency		16.44 %	6.08
				Payback percentage		91.10 %	

When playing four cards on Lucky Clover, the four cards are independent of one another. Each card has a hit frequency of 16.44 %, which means that 83.56 % of plays on a card are losses. We defined the hit frequency as a win on at least one card (i.e. LDWs are included as hits because players typically misinterpret them as wins). So if one were to put wagers on four cards then it would be a hit if 1, 2, 3, or 4 of the cards has a win of any size. We calculated the hit frequency broken down by cards wagered and the results are that playing 1 card has a hit frequency is 16.44 %, for 2 cards it is 20.18 %, for 3 it is 41.66 %, and over half of the outcomes when playing all 4 cards will be a win or LDW (51.25 %).

We wrote a computer program to simulate the playing of one million cards on *Lucky Clover*. The payback percentage we obtained was 91.94 %, which means an estimated hold of 9.06 %, which differs only slightly due to sampling error from the stated hold of the game which is 8.90 % (see Table 1). In the program we considered the 1,000,000 outcomes as if the player were wagering on four cards at a time, thus yielding 250,000 plays. The 250,000 plays in our simulation had a hit frequency of 51.34 % broken down as follows:

- 48.66 % regular losses
- 19.13 % LDWs
- 32.21 % regular wins

The manner in which the balls are drawn in *Lucky Clover* is different than in regular/live bingo. In regular bingo, the 75 balls (numbered 1–75) are in one physical drum, and balls are randomly drawn one at a time. The numbers on *Lucky Clover* are drawn randomly but from five separate arrays, as if from five different drums, with one drum for each of the five letters in B-I-N-G-O. In *Lucky Clover* there are always five balls drawn between 1 and 15, five between 16 and 30, four between 31 and 45, five between 46 and 60, and five between 61 and 75. The player is not informed about this difference between *Lucky Clover* and regular bingo. When the 24 balls are shown to the player, as in Fig. 1, they are presented in random order so it is not obvious to the player that the balls are drawn differently than in regular bingo. No explanation is provided as to why the balls are drawn differently than in regular bingo but displayed randomly as in regular bingo.

### Fast and continuous play

Like slot machines, *Lucky Clover* and other POD Bingo games can be played at a much faster rate than paper Bingo. Players are able to complete a play in as little as 2 s, or 30 plays per minute if playing continuously. The high frequency of small wins and LDWs reinforces continued play. For example, assume a player arrives with $20 and plays until broke by betting $1.00 per play with $0.25 on each of four *Lucky Clover* cards. That player may lose an average of 8.90 cents per spin because the hold of the game is 8.90 %, and it may take an average of 225 spins (20 divided by $0.089) for the player to go broke. Such a session would have the player wagering a total of $225 and at 30 plays per minute it would take approximate 7.5 min for the player to go broke. The player’s original bankroll of $20 is only a small fraction of the total amount wagered. The other $205 is won mostly as LDWs and small wins, with perhaps a few larger wins. With wagers placed on four cards, the hit frequency in *Lucky Clover* is 51.34 %, so the player would have an average of 116 positively reinforcing hits (225 * 51.34 % equals 116), made up of an expected 73 regular wins and 43 LDWs—even though the player ends up going broke.

### Players control the frequency and size of wins

Our computer simulation of *Lucky Clover* found that players have the choice to increase the hit frequency from 16.44 % with one card played up to 51.34 % with four cards, and that approximately 19.13 % of plays result in an LDW when playing all four cards. Although LDWs are obviously losses, the celebratory sights and sounds that occur during play serve to camouflage this fact (Dixon et al. 2015a) and may positively reinforce continued play (Dixon et al. 2010; Wilkes et al. 2010). The choice of how many cards per play gives the player the ability to control how often they ‘win’, much like slots players can control the hit frequency by their choice of the number of paylines (Harrigan et al. 2011). They can also adjust the nominal size of prizes by a factor of 4 by betting from $0.25 up to $1.00 per card. Giving players control over these parameters has no effect on the hold, but may nevertheless give players a sense that they can improve their odds of turning a profit if they play skilfully. These superfluous controls are therefore likely to support cognitive distortions such as illusion of control and gambler’s fallacy that are common among problem gamblers (Goodie and Fortune 2013).

### Near misses

Near misses in games of chance are objectively equivalent to an outright miss, but appear similar to wins and subjectively motivate continued risk taking. This may happen in slots games by heightening the anticipation of imminent reward (Clark et al. 2013; Dixon et al. 2013), by increasing excitement and physiological arousal (Dixon et al. 2011), and possibly by suggesting to the player that they are becoming skilled at the game (Griffiths 1991; Reid 1986). The fact that *Lucky Clover* highlights the near-misses with flashing red graphics means that players are frequently reminded of near-misses, which occur on over 30 % of cards.

### List of past wins

*Lucky Clover* provides the player with a list of the past ten wins, but not the losses. This feature increases the salience of past wins compared to past losses and makes the wins more memorable (Scoboria and Wilson 2011). Problem gamblers might use this information in their vain attempts to predict future outcomes according to distorted heuristics like the gambler’s fallacy (Tversky and Kahneman 1974) or hot hand fallacy (Gilovich et al. 1985). The game could easily be designed not to have this listing of wins, and its only purpose appears to be to stimulate poor decision making among players who have a tendency toward cognitive distortions that are typical of problem gamblers.

## Does POD Bingo Pose Potential Harm to Players?

We have observed that computerized POD Bingo games have some key structural characteristics that are similar to those of multiline slots games. This is of concern because the context into which these new games are being introduced in Ontario Charitable Gaming Centres makes them likely to increase the incidence of problem gambling among an already well-entrenched community of Bingo enthusiasts. According to the Pathways Model of Problem and Pathological Gambling (Blaszczynski and Nower 2002), the most important prerequisite for the emergence of problem gambling is the availability and access to gambling. In this case, potentially habit-forming games are being made available to existing Bingo players. One peculiarity of the introduction of POD games into Charitable Gaming Centres is that they are being marketed to young people more than other forms of electronic gambling becasue the minimum age to enter the Charitable Gaming Centres is 18 years whereas they must be 19 years old to enter casinos and slots-at-racetracks in the province. This is problematic because of the high participation of young people in Bingo (Moubarac et al. 2010), and because of the high rates of problem gambling among young people (Lussier et al. 2009).

Bingo halls have existed in Ontario for decades and are socially accepted in large part because they provide funding to charities. In the terminology of the Pathways Model, this public acceptance is an ecological determinant that relates to “…public policy and regulatory legislation that create and foster an environment in which gambling is socially accepted, encouraged and promoted.” (p. 491). Advertisements by OLG with slogans such as “When you revitalize bingo centres in Ontario, your local charities win.” (*National Post*, October 9, 2012) emphasize the social benefits of fund raising. They fail to mention that the co-location of traditional paper-based Bingo beside the new electronic forms of the game, as well as slots-like video instant ticket dispensers, is potentially far more dangerous to players than the general public might realize.

The structural characteristics of POD Bingo games are similar to the structural characteristics of multiline slot machines. Both are designed to provide an experience of fast and continuous play, during which players control the frequency and size of wins with the reinforcement rate exaggerated by LDWs, and to enhance the anticipation of imminent reward by emphasizing near miss outcomes. Some of the games we examined also list recent winning outcomes and have bonus rounds similar to those seen in most multiline video slots games. Once again, the Pathways Model would predict that any game with these structural characteristics may be a potential source of problem gambling due to “the influence of classical and operant conditioning leading to increased participation and the development of habitual patterns of gambling, and cognitive process resulting in faulty beliefs related to personal skill and probability of winning” (p. 491). Thus the modernization of charitable gaming in Ontario meets all three of the common processes in the Pathways Model of Problem and Pathological Gambling. Bingo players who have personal characteristics that make them susceptible to problem gambling (e.g. high impulsivity), might be more likely to realize that unfortunate potential if they partake in these more intense electronic forms of the game. We conclude that rates of problem gambling and other harms should be carefully monitored during and after the rollout of these new Bingo EGMs in Ontario, as well as in other jurisdictions that introduce this kind of gambling to their charitable or other gaming sectors. Careful monitoring is necessary to determine if future steps can be taken to minimize harm, while ensuring the safe and socially responsible modernization of these enterprises.
